# Gefitinib Inhibits Invasive Phenotype and Epithelial-Mesenchymal Transition in Drug-Resistant NSCLC Cells with *MET* Amplification 

**DOI:** 10.1371/journal.pone.0078656

**Published:** 2013-10-22

**Authors:** Silvia La Monica, Cristina Caffarra, Francesca Saccani, Elena Galvani, Maricla Galetti, Claudia Fumarola, Mara Bonelli, Andrea Cavazzoni, Daniele Cretella, Rita Sirangelo, Rita Gatti, Marcello Tiseo, Andrea Ardizzoni, Elisa Giovannetti, Pier Giorgio Petronini, Roberta R. Alfieri

**Affiliations:** 1 Department of Clinical and Experimental Medicine, University of Parma, Parma, Italy; 2 Department Medical Oncology, VU University Medical Center, Amsterdam, The Netherlands; 3 Italian Workers' Compensation Authority (INAIL) Research Center at the University of Parma, Italy; 4 Department of Biotechnology, Biomedical and Translational Sciences, University of Parma, Parma, Italy; 5 Division of Medical Oncology, University Hospital of Parma, Parma, Italy; Seoul National University, Korea, Republic Of

## Abstract

Despite the initial response, all patients with epidermal growth factor receptor (EGFR)-mutant non-small cell lung cancer (NSCLC) eventually develop acquired resistance to EGFR tyrosine kinase inhibitors (TKIs). The EGFR-T790M secondary mutation is responsible for half of acquired resistance cases, while *MET* amplification has been associated with acquired resistance in about 5-15% of NSCLCs. Clinical findings indicate the retained addiction of resistant tumors on EGFR signaling. Therefore, we evaluated the molecular mechanisms supporting the therapeutic potential of gefitinib maintenance in the HCC827 GR5 NSCLC cell line harbouring *MET* amplification as acquired resistance mechanism. We demonstrated that resistant cells can proliferate and survive regardless of the presence of gefitinib, whereas the absence of the drug significantly enhanced cell migration and invasion. Moreover, the continuous exposure to gefitinib prevented the epithelial-mesenchymal transition (EMT) with increased E-cadherin expression and down-regulation of vimentin and N-cadherin. Importantly, the inhibition of cellular migration was correlated with the suppression of EGFR-dependent Src, STAT5 and p38 signaling as assessed by a specific kinase array, western blot analysis and silencing functional studies. On the contrary, the lack of effect of gefitinib on EGFR phosphorylation in the H1975 cells (EGFR-T790M) correlated with the absence of effects on cell migration and invasion. In conclusion, our findings suggest that certain EGFR-mutated patients may still benefit from a second-line therapy including gefitinib based on the specific mechanism underlying tumor cell resistance.

## Introduction

Non-small cell lung cancer (NSCLC) is the leading cause of cancer death in the world, and traditional chemotherapeutic drugs have only a palliative effect [1]. However, the discovery of epidermal growth factor receptor (EGFR) activating mutations and the response to EGFR tyrosine kinase inhibitors (TKIs), such as gefitinib and erlotinib, deeply changed the management of advanced NSCLC in the last decade [2–4]. Small in-frame deletions in exon 19 and point mutations within exon 21 (L858R) are the most common EGFR activating mutations both leading to sustained activity of the kinase. These mutations increase the susceptibility to EGFR-TKIs activity [5,6] but all the EGFR-mutant lung cancer patients experience disease progression within 10 to 14 months from the beginning of the therapy [7–9]. Various mechanisms of resistance have been identified [10]. The acquisition of the EGFR-T790M secondary mutation is responsible for half of the cases of acquired resistance to EGFR-TKIs [11,12] and *MET* amplification, allowing cell survival by persistent Akt signaling activation, has been described for 5 to 15% of cases [13–15]. PIK3CA mutations and transformation to SCLC have also been implicated as mechanisms of resistance to EGFR-TKIs [16]. Furthermore, recent studies reported that the epithelial-mesenchymal transition (EMT), a process in which cells lose their epithelial features and acquire a mesenchymal fibroblastoid phenotype enhancing their motility and invasion capability, might also play a role in the development of resistance to EGFR-TKIs in NSCLC [17,18]. 

TKI-resistant NSCLC patients are commonly treated with chemotherapeutic drugs. However, several clinical indications suggest that EGFR-mutant lung cancers maintain partial sensitivity to TKIs despite development of acquired resistance and tumors can still be sensitive to EGFR-TKIs treatment beyond progression [19–22] or re-treatment at further progression [23–25]. In addition Chaft and collaborators documented that in a series of patients discontinuing EGFR-TKI prior to enrolling in a clinical trial for acquired resistance, 22% developed accelerated progression leading to hospitalization, occurring after a median of 8 days [26].

Novel strategies under investigation include the continuation beyond progression of EGFR-TKIs combined with chemotherapy, the re-challenge with TKIs after second-line chemotherapy, the use of irreversible TKIs or the combination with novel agents targeting different molecular pathways. 

Further preclinical studies to describe molecular mechanisms and potential markers of drug activity are also warranted. Therefore, in this study we explored the retained antitumor activity of gefitinib in resistant HCC827-GR5 and NCI-H1975 NSCLC cells, carrying *MET* amplification and T790M mutation, respectively.

Our results indicate that HCC827 GR5 cells can proliferate and survive regardless of the presence of gefitinib, whereas its absence enhanced their migrating and invading capabilities and allowed the acquisition of mesenchymal markers (down-regulation of E-cadherin and up-regulation of vimentin and N-cadherin). The maintenance of gefitinib, instead, reduced cell migration, invasion and allowed the maintenance of an epithelial phenotype. On the contrary, in H1975 cells, the gefitinib treatment had no effect either on cell proliferation, migration or invasion.

These results suggest that patients who are more likely to benefit from continuing gefitinib treatment after tumor progression may be selected based on the mechanisms of acquired resistance to the first-line treatment with EGFR-TKIs.

## Materials and Methods

### Cell Culture

Human HCC827 GR5 NSCLC cell line was kindly provided by Dr P. Jänne (Dana-Farber Cancer Institute, Boston MA) and it was obtained from gefitinib-sensitive EGFR exon 19 mutant HCC827 cell line by exposing these cells to increasing concentration of gefitinib for 6 months as previously described [13]. Calu-3 and NCI-H1975 (H1975) were from ATCC (Manassas, VA). Cells were cultured as recommended and maintained under standard cell culture conditions at 37°C in a water-saturated atmosphere of 5% CO_2_ in air. HCC827 GR5 cells were cultured in the presence of gefitinib 1μM.

### Drug treatment

Gefitinib (ZD1839/Iressa^®^) was provided by AstraZeneca (Milan, Italy). NVP-BEZ235 was provided by Novartis Institutes for BioMedical Research (Basel, Switzerland). Dasatinib was from LC Laboratories (Woburn, MA). U0126 and SU11274 were from Sigma-Aldrich (St. Louis, MO). PD168393 was from Calbiochem (La Jolla, CA). BIBW2992 (afatinib) was from inpatient pharmacy. Drugs were dissolved in DMSO (Sigma) and diluted in fresh medium before use. Final DMSO concentration in medium never exceeded 0.1% (v/v) and equal amounts of the solvent were added to control cells.

### Analysis of cell proliferation, cell death and cell cycle

Cell proliferation was evaluated by cell counting in a Bürker hemocytometer by trypan blue exclusion and by crystal violet staining as previously described [27]. Cell viability was evaluated by tetrazolium dye [3-(4,5-dimethylthiazol-2-yl)-2,5-diphenyltetrazolium- bromide] (MTT) assay as previously described [28]. Data are expressed as percent inhibition of cell proliferation versus control cells. Cell death was assessed on stained (Hoechst 33342/PI) cells using fluorescence-microscopy. Distribution of the cells in the cell cycle was determined by PI staining and flow cytometry analysis as described elsewhere [29]. 

### Wound healing assay

A wound-healing assay was performed with the CytoSelect 24-well Wound Healing Assay Kit (Biolabs, San Diego, CA,). Wound healing inserts were put into 24-well cell culture plates and cell suspension (1.5x10^5^ cells in 250 μl) was added to either side of the insert and incubated overnight to form a monolayer. The inserts were then removed to allow the cells to migrate. After 24 hours cells were fixed with 100% methanol, stained with hematoxylin. Images of wound healing were captured by microscope equipped with digital camera at a magnification of ×40 at zero time point and after 24h. Cell migration was quantified by measuring the migration distances. Percent closure was calculated as wound area 24h/wound area zero time point x 100. 

### Cell migration and invasion

The migration and invasion assays were carried out using Transwell chamber with 6.5-mm diameter polycarbonate filters (8μm pore size, BD Biosciences, Erembodegem, Belgium) uncoated or coated with Matrigel^TM^. Cells were trypsinized and 2x10^5^ cells suspended in serum free RPMI-1640 medium and loaded in the upper wells. FBS (10%) was used as a chemoattractant in the lower chambers. After incubation for 16 hours all of the non-migrated (or non-invaded) cells were removed with a cotton swab, and cells that have migrated (or invaded) through the membranes were fixed with 100% methanol, stained with hematoxylin and counted under a Phase contrast microscope. 

### Gelatine zymography

The gelatin zymography was performed to determine the activity of matrix metalloproteinases (MMP). Equal number of cells were seeded and incubated with serum-free RPMI-1640 for 24h in the presence or in the absence of gefitinib 1 µM. Medium was collected and centrifuged at 1800 rpm for 5 min to remove cell debris. Equal amounts of media were mixed with SDS-PAGE sample buffer 4X in the absence of reducing agent and electrophoresed in 10% polyacrylamide gel containing 1mg/ml gelatin. After running, the gel was incubated in the Renaturating Buffer (50mM Tris-HCl pH 7, 6.5mM CaCl_2_, 1µM ZnCl_2_, 2.5% Triton X-100) two times for 15 min at room temperature. The gel was washed with Washing Buffer (50mM Tris-HCl pH 7, 6.5mM CaCl_2_, 1µM ZnCl_2_) and then it was incubated in Developing Buffer (50 mM Tris-HCl pH 7, 6.5 mM CaCl_2_, 1µM ZnCl_2_, 1% Triton X-100, 0.02% NaN_3_) overnight at 37°C. The gel was stained with 0.25%. Coomassie Brilliant Blue R-250 solution containing 45% methanol and 10% glacial acetic acid for 4 hours and then washed with a solution containing 10% glacial acetic acid, 45% methanol for 3 hours. Areas of protease activity appeared as clear bands. The activity of MMPs was determined by densitometric scanning of the bands and analysis by Quantity One 1-D Analysis Software (BIO-RAD, Hercules, CA).

### Soft agar assay

Cells were suspended in RPMI1640 containing 0.3% low melting agarose, and plated onto solidified 0.5% agar containing RPMI1640 in six-well culture plates at a density of 10000 cells per well. Cells were incubated at 37°C in 5% CO_2_ incubator and once a week fresh culture medium was added in each well. After 4 weeks colonies were stained with 0.005% crystal violet and quantified under a phase contrast microscope. 

### Western blot analysis

Procedures for protein extraction, solubilization, and protein analysis by 1-D PAGE are described elsewhere [28]. Antibodies against p-EGFR^Tyr1068^, EGFR, MET, p-Src^Tyr416^, p-Src^Tyr527^, Src, p-Akt^Ser473^, Akt, p-p70S6K^Thr389^, p70S6K, p-ERK1/2, ERK1/2, p-p38 MAPK, p38 MAPK; p-STAT5^Tyr694^, STAT5, E-cadherin, N-cadherin, vimentin, SNAIL, SLUG were from Cell Signaling Technology (Beverly, MA); antibody against p-MET^Tyr1234/1235^ was from Upstate (Lake Placid, NY); antibody against GAPDH was from Ambion (Austin, TX). HRP-conjugated secondary antibodies were from Pierce (Rockford, IL) and chemoluminescence system (Immobilion^TM^ Western Chemiluminescent HRP Substrate) was from Millipore (Millipore, Temecula, CA). Reagents for electrophoresis and blotting analysis were from BIO-RAD. 

### Determination of pattern of protein phosphorylation

Relative levels of phosphorylation of 46 kinase phosphorylation sites (38 selected proteins) were obtained by using Proteome Profiler Human Phospho-kinase Array (Kit ARY003B from R&D System, Minneapolis, MN) according to the manufacturer’s guidelines. A total of 300μg of proteins was used for each array. The resulting spots were quantified using Quantity One 1-D Analysis Software (BIO-RAD). 

### RNA interference assay

Cells were transfected with Invitrogen Stealth^TM^ siRNA (Invitrogen, Carlsbad, ca) against: EGFR (mixture of HSS103114, HSS103116 and HSS176346) with a final concentration of 60nM; Src (mixture of HSS186080, HSS186081 and HSS186082) with a final concentration of 60nM; p38α (mixture of HSS102352, HSS102353 and HSS175313) with a final concentration of 60nM; STAT5a/b (mixture of HSS186133, HSS186134, HSS186135, HSS110287, HSS110288 and HSS110289) with a final concentration of 90 nM. Negative controls (medium GC content and low GC content) were from Invitrogen. The transfection was carried out according to the Invitrogen forward transfection protocol for Lipofectamine^TM^ RNAiMAX transfection reagent. After 48 hours of transfection, medium was aspirated and replaced with exposure medium. 

### Immunofluorescent staining

Cells were grown on poly-L-lysine–coated glass slides for 24h. For E-cadherin staining, cells were fixed with 4% formaldehyde in PBS for 20min and unspecific epitopes were blocked with 3% BSA in PBS. Then, cells were incubated for 3h at RT with the anti-E-cadherin antibody (Cell Signaling Technology). For vimentin staining, cells were fixed in with 4% paraformaldehyde in PBS for 15min, washed with PBS, permeabilized with 0.2% Triton X-100 at RT for 30min and blocked with 3% BSA. Then, cells were incubated overnight at 4°C with anti-vimentin antibody (Cell Signaling Technology). For E-cadherin and vimentin stainings secondary antibodies FITC-conjugated donkey IgG anti-rabbit (Jackson Immunoresearch, West Grove, PA). were used. Nuclei were stained with Draq5 (Cell Signaling Technology). Samples were observed using a confocal system (LSM 510 Meta scan head integrated with the Axiovert 200 M inverted microscope; Carl Zeiss, Jena, Germany) with a X63 oil objective. Image acquisition was carried out in multitrack mode, namely through consecutive and independent optical pathways.

### Quantitative Real-Time PCR

Total RNA was isolated using the TRI REAGENT LS (Invitrogen). One µg RNA was retro-transcribed using the DyNAmo cDNA Synthesis Kit (Thermo Scientific, Vantaa, Finland), according to the manufacturers’ instructions. Primers and probes to specifically amplify vimentin were obtained from Applied Biosystems Assay-on-Demand Gene expression products (Hs00185584_m1). The quantitative real-time PCR was performed in a 25-μl reaction volume containing TaqMan Universal master mix (Applied Biosystems, Forster City, CA). All reactions were performed in triplicate using the ABI PRISM 7500 sequence detection system instrument (Applied Biosystems). Samples were amplified using the following thermal profile: 50°C for 2 min, 95°C for 10 minutes, 40 cycles of denaturation at 95°C for 15 sec followed by annealing and extension at 60°C for 1 minute. Amplifications were normalized to GAPDH (Hs02758991_g1). The fold change was calculated by the ΔΔC_T_ method and results were plotted as 2 ^-ΔΔCT^.

### Statistical analysis

Statistical analyses were carried out using GraphPad Prism version 5.00 software (GraphPad Software, San Diego, CA). Results are expressed as mean values ± standard deviations (SD) for the indicated number of independent measurements. Differences between the mean values recorded for different experimental conditions were evaluated by Student’s t-test, and P values are indicated where appropriate in the figures and in their legends. Significance of difference is indicated as ***P<0.001, **P<0.01 and *P<0.05.

## Results

### Gefitinib inhibits cell migration, invasion and anchorage independent growth in HCC827 GR5 cells

As previously reported [13] and independently confirmed in our laboratory (data not shown), the gefitinib-resistant clone HCC827 GR5 showed a 1000-fold higher IC_50_ than the parental cell line HCC827 (10μM versus 10nM, respectively) and is characterized by amplification of the *MET* oncogene, leading to ERBB3-mediated activation of PI3K/AKT signaling. Src protein activation was also markedly increased supporting a key role of Src signaling in gefitinib-resistance, as reported previously [30].

**Figure 1:**
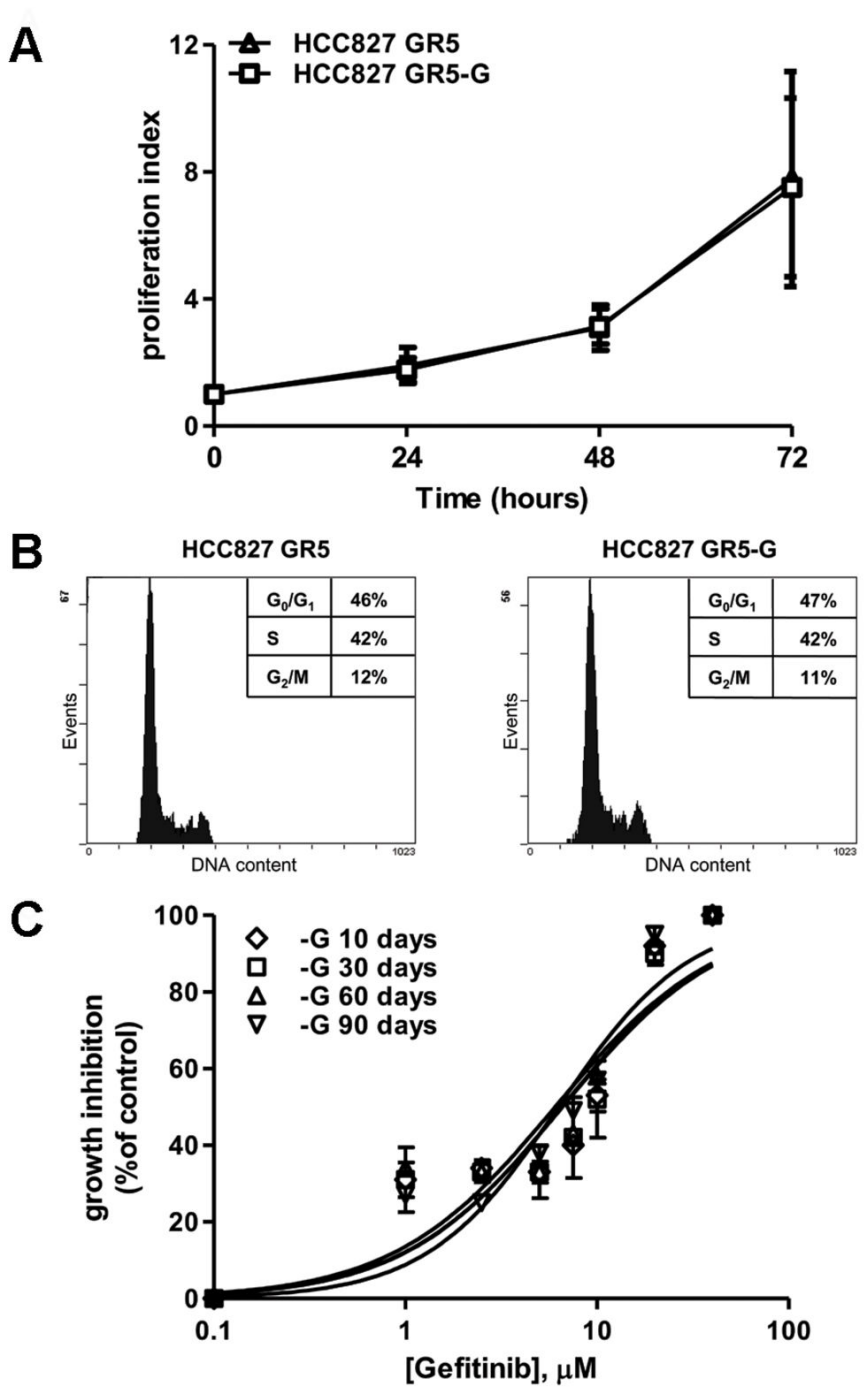
Effect of gefitinib withdrawal on cell proliferation. (A) HCC827 GR5 grown in the presence of 1 µM gefitinib and HCC827 GR5-G (maintained in the absence of gefitinib for 10 days) cells were seeded in a 96-multiwell plate. After 24, 48 and 72h cell proliferation was assessed using crystal violet staining; cell proliferation index was calculated as the ratio between the OD at each point time and the OD at zero time point. Mean values of three independent measurements (±SD) are shown. (B) 24h from seeding, HCC827 GR5 and HCC827 GR5-G were stained with propidium iodide and analyzed by flow cytometry. Cytofluorimetric profiles and percentages of cells residing in each cycle phase are from a representative experiment of three independent experiments. (C) HCC827 GR5 cells gefitinib-deprived for the indicated period of time were exposed for 72h to different concentrations of gefitinib (1 to 40 µM) and then cell growth was assessed using MTT assay. Data are expressed as percent inhibition of cell proliferation versus control cells and are means (±SD) of three independent experiments.

After a 10 days gefitinib removal the HCC827 GR5 cells did not modify either the proliferation index (Figure 1A) or the cell cycle distribution (Figure 1B) as compared to HCC827 GR5 cells grown in the presence of 1μM gefitinib. Comparable results were obtained after 30 days of gefitinib removal (not shown). Moreover, the absence of gefitinib did not alter HCC827 GR5 resistance to gefitinib for up to 90 days of deprivation. Indeed, as shown in Figure 1C, cells deprived of gefitinib for different periods of time and acutely treated with the drug for 72h still maintained the resistant phenotype (IC50>7μM). The 20-30% of cell growth inhibition observed at 1μM might be ascribed to the presence of a small sub-population of previously quiescent gefitinib-sensitive cells that during the period of gefitinib deprivation might expand and subsequently arrested when re-exposed to the drug. The restoration of drug sensitivity after EGFRTKI withdrawal has been previously reported by Chmielecki et al. in T790M resistant PC9 cells after multiple passages without the inhibitor [31].

**Figure 2:**
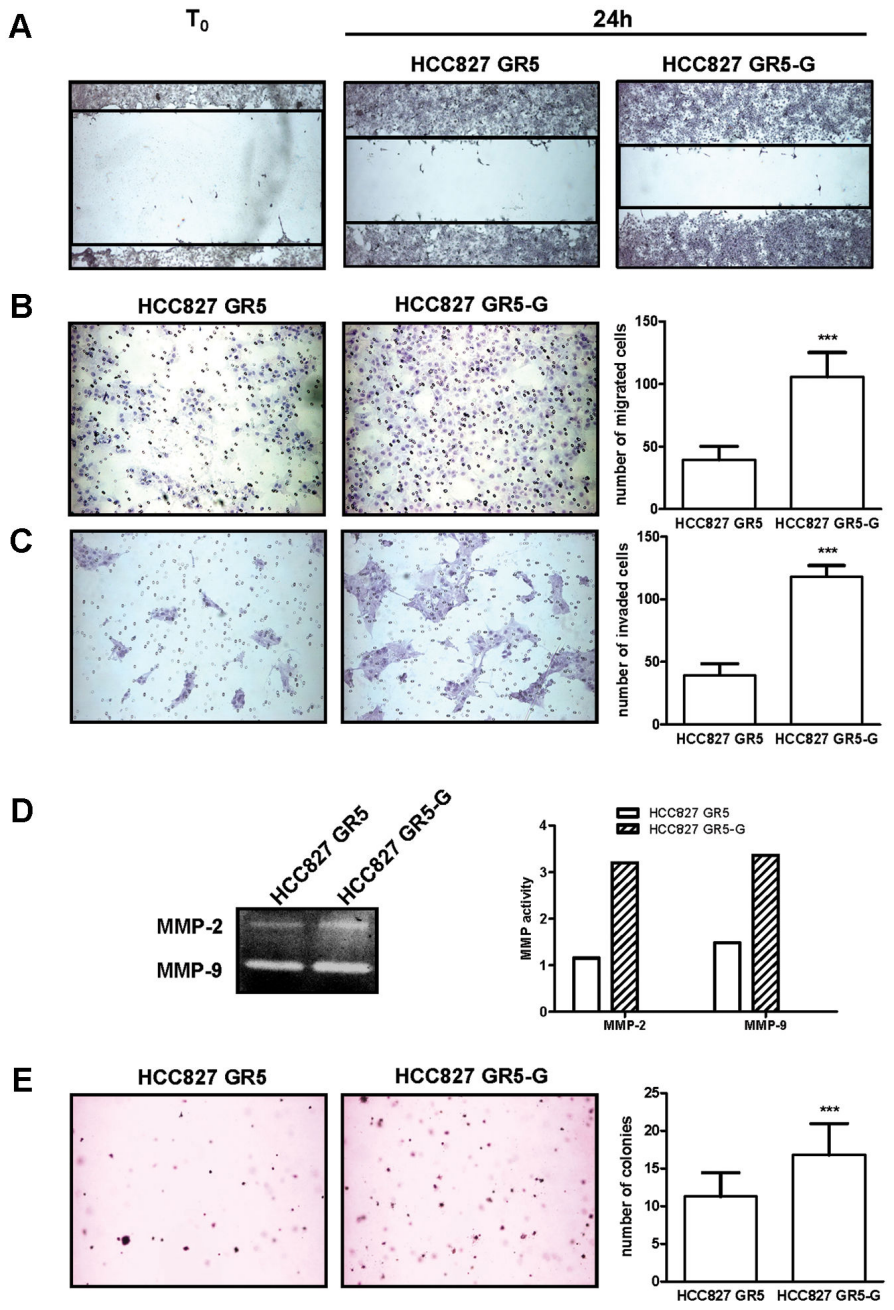
Effect of gefitinib withdrawal on cell migration, invasion and growth on soft agar. Wound-healing, migration, invasion, and soft agar colony assays were performed on HCC827 GR5 grown in the presence of 1 µM gefitinib and HCC827 GR5-G (maintained in the absence of gefitinib for 10 days) cells. (A) Representative images of the wound tracks were obtained at time point zero and after 24 hours (magnification of 40X). Representative fields of migration (B) or invasion (C) are shown (magnification of 100X). Columns, means of 10 fields counted; bars, SD. (D) Gelatin zymography analysis of media from HCC827 GR5 and HCC827 GR5-G incubated with serum-free medium for 24h. Columns, enzyme activity of MMP-2 and MMP-9 determined by densitometric analysis. (E) Representative fields of colony formation are shown (magnification of 40X). Columns, means of 10 fields counted; bars, SD. Results are representative of three independent experiments. ***P<0.001.

HCC827 GR5 cells cultured in the absence of gefitinib for 10 days showed a significant increase in both cell migration and invasion as detected by wound healing and Boyden chambers assay. HCC827 GR5 cells spread into the wound area more efficiently than cells continuously exposed to gefitinib, with wound closure percentages of 48%±4.2 and 37%±3.2 respectively (P<0.01, Figure 2A). Moreover, as shown in Figure 2B, the HCC827 GR5 gefitinib-deprived, compared to HCC827 GR5 cells cultured with gefitinib, exhibited 2.7-fold increase in the number of migrating cells through the uncoated PET membrane in Boyden chambers. Furthermore, we observed a 3-fold increase in invading cells through the Matrigel-coated PET membrane for the HCC827 GR5 gefitinib-deprived cells (Figure 2C). Considering the role of matrix metalloproteinases (MMPs) in degrading extracellular matrix components, the effect of gefitinib removal on the proteolytic activity of MMP-2 and MMP-9 was evaluated by using a gelatin zymography assay showing an approximately 2 fold increase of MMP-2 and MMP-9 activity in cells deprived of gefitinib for 10 days. These results indicate that the maintenance of gefitinib inhibited secretion and activation of gelatinolytic MMP-2 and MMP-9 (Figure 2D).

The effect of gefitinib withdrawal on the anchorage-independent growth was also examined by using the soft agar colony assay. The number of anchorage-independent colonies of the cells cultured in the absence of gefitinib was significantly increased as compared to the gefitinib-treated cells (Figure 2E).

### Gefitinib inhibits signal transduction pathway involved in cellular motility

**Figure 3:**
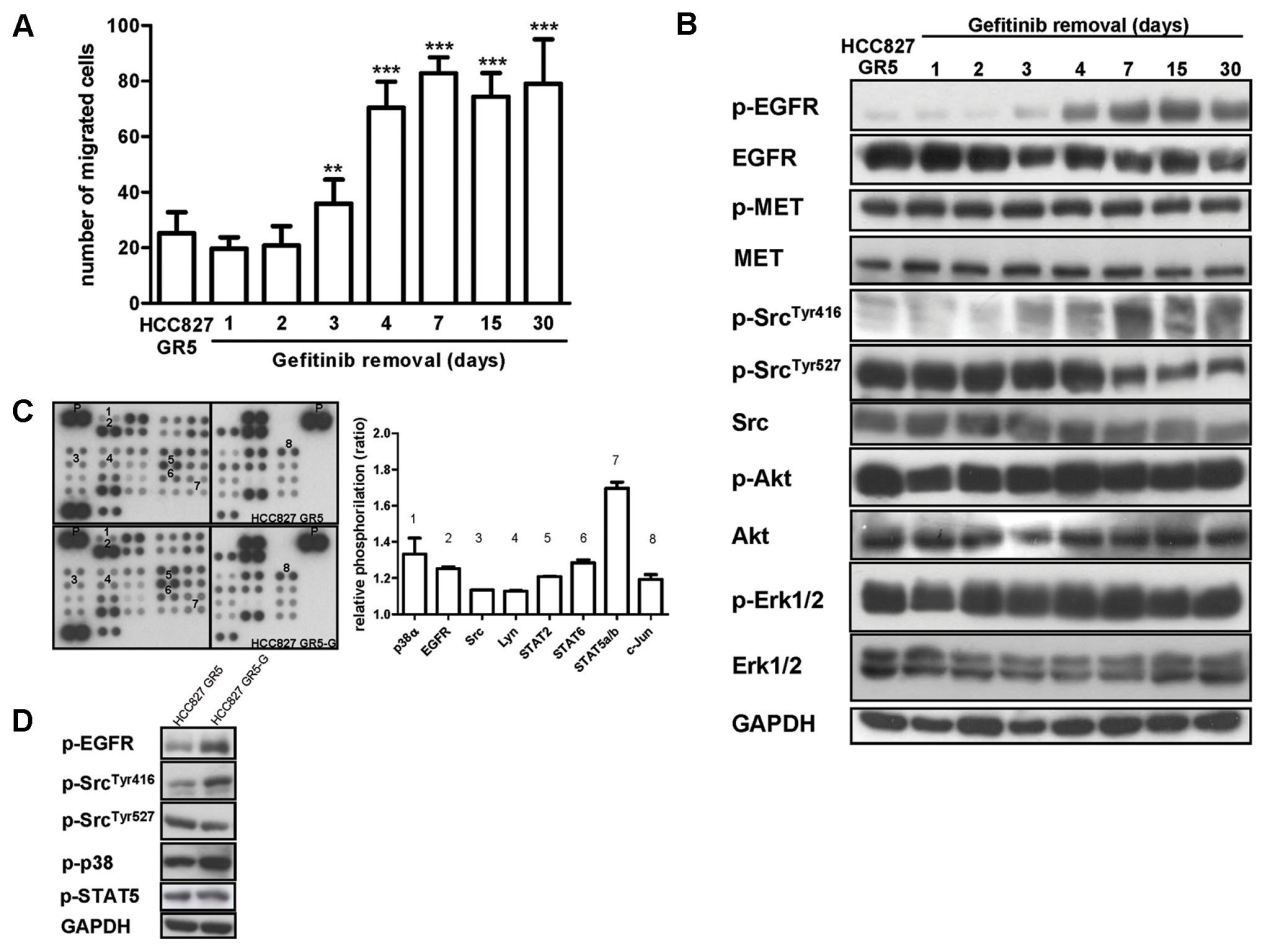
Effect of gefitinib withdrawal on signal transduction pathways. HCC827 GR5 cells were deprived of gefitinib for 1, 2, 3, 4, 7, 15 or 30 days. (A) Migration assay was performed at each time point. Columns, means of 10 field counted; bars, SD. ***P<0.001, **P<0.01. (B) Expression of the indicated proteins was analyzed by Western blotting at each time point. Results are representative of three independent experiments. (C) HCC827 GR5 grown in the presence of 1 µM gefitinib and HCC827 GR5-G (maintained in the absence of gefitinib for 7 days) lysates were incubated with human phospho-kinase array membranes and bound phospho-proteins were detected according to kit instructions. Each membrane contains specific kinase and positive control antibodies (P) spotted in duplicate. Columns, means of relative levels of protein phosphorylation (ratio of phosphorylation of HCC827 GR5-G/ HCC827 GR5 cells) of duplicate spots 1-9 from a single experiment; bars, SD. Eight proteins (EGFR, p38α, Src, Lyn, STAT2, STAT6, STAT5a/b and c-Jun) exhibited a significant increase (P<0.05) in their phosphorylation status following gefitinib removal (D). Lysates were analyzed by Western blotting using the indicated antibodies.

Increased motility was observed in HCC827 GR5 cells after 3 days of gefitinib withdrawal (Figure 3A). A plateau of cellular migration was reached after 7 days and this phenomenon correlates with the increase in EGFR, and Src^Tyr416^ phosphorylation (Figure 3B). Moreover, we observed a marked reduction in Src^Tyr527^ phosphorylation, which negatively regulates Src kinase activity after 7 days since gefitinib withdrawal. By contrast, no differences were detected for MET, Akt and ERK 1/2 phosphorylation status. To better investigate the signaling pathways activated after 7 days of gefitinib removal, 43 specific Ser/Thr or Tyr phosphorylation sites of 35 different proteins were analyzed by a human phospho-kinase array kit. Eight proteins, including p38α, EGFR, Src^Tyr416^, Lyn, STAT2, STAT6, STAT5a/b and c-Jun, exhibited a significant increase (p<0.05) in their phosphorylation status following gefitinib removal (Figure 3C). The increased phosphorylation of p38, STAT5 and Src was validated by Western blotting in cells deprived of gefitinib for 7 days (Figure 3D). 

### EGFR modulation correlates with gefitinib-related regulation of cell motility

**Figure 4:**
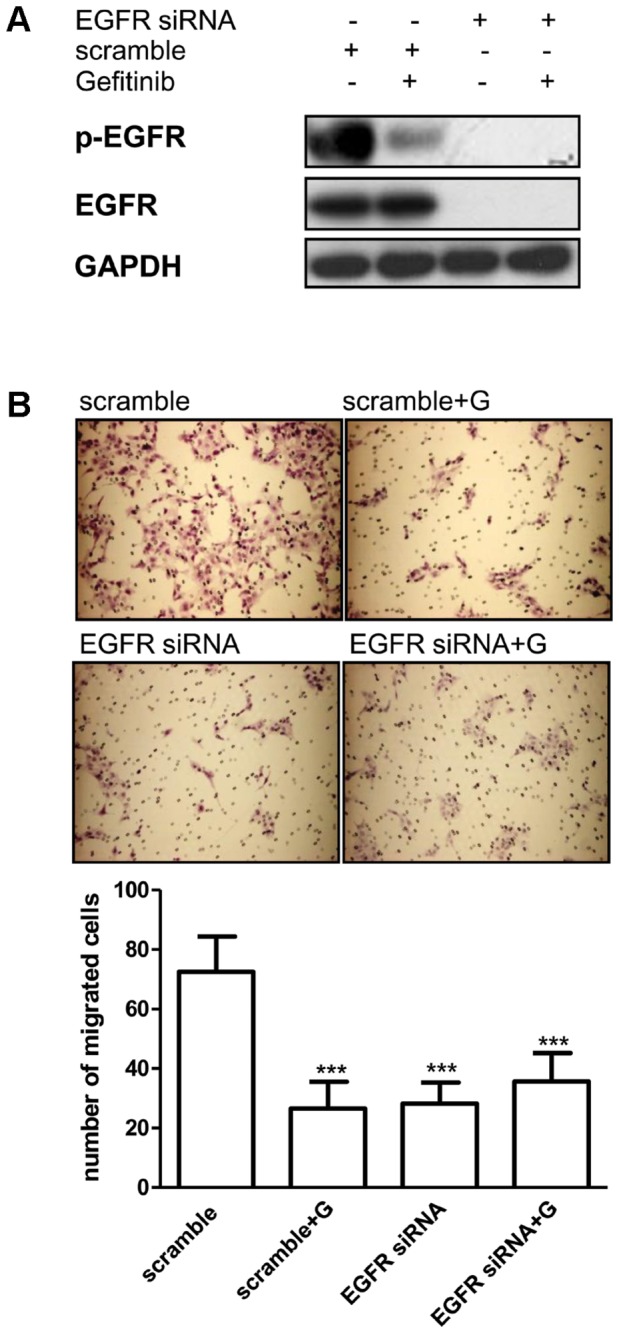
Effect of EGFR silencing on cell migration. HCC827 GR5-G (maintained in the absence of gefitinib for 10 days) were transfected with EGFR siRNA or control siRNA (scramble) for 48 h. Then medium was replaced with fresh medium with or without gefitinib 1μM for 16h and the expression of the indicated proteins was analyzed by Western blotting (A) or cells were seeded on culture inserts in the absence or in the presence of 1μM gefitinib for migration assay (B). Representative fields of migration are shown (magnification of 100X). Columns, means of 10 fields counted; bars, SD. Results are representative of three independent experiments. ***P<0.001.

To assess the role of EGFR re-activation in the acquisition of migratory capability, HCC827 GR5 cells maintained for 10 days in the absence of gefitinib were transfected with EGFR-specific or scramble siRNAs. EGFR expression was verified by Western blotting 72 hours post-transfection (Figure 4A). One μM gefitinib was added, where indicated, 24 hours before blotting. As expected, siRNA- EGFR completely inhibited EGFR expression compared to the negative siRNA-scramble control. As shown in Figure 4B, the inhibitory effect on cell migration obtained by silencing EGFR in HCC827 GR5 cells was similar to the one observed in the presence of gefitinib. Moreover, the addition of gefitinib to siRNA-EGFR transfected cells did not further decrease the amount of migrating cells. These results demonstrated the dependency of HCC827 GR5 cells motility on EGFR activity and suggested that the observed effect of gefitinib on cell migration is associated with the inhibition of its target.

**Figure 5:**
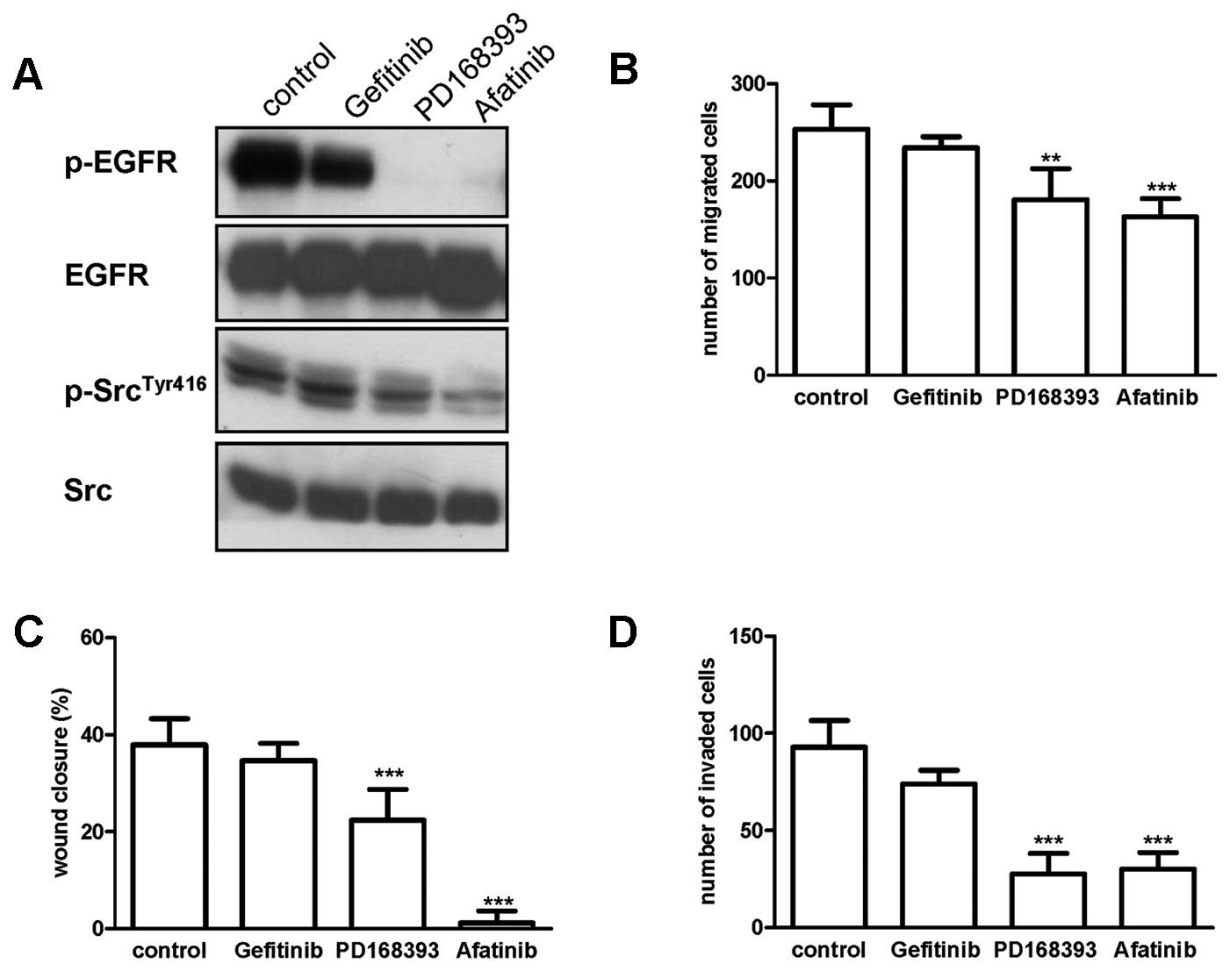
Effect of EGFR inhibition on cell migration and invasion of H1975 cells. (A) H1975 cells were treated with 1μM gefitinib, PD168393 or afatinib. After 5h protein expression was assessed by Western blotting using the indicated antibodies. (B) H1975 cells were seeded on culture inserts in the absence or in the presence of 1μM EGFR inhibitors for (B) migration and (D) invasion assay. (C) Wound-healing assay was performed on H1975 cells in the presence of 1μM EGFR inhibitors. Columns, means of 10 fields counted; bars, SD. Results are representative of three independent experiments. ***P<0.001, **P<0.01.

To confirm the involvement of EGFR in controlling cell migration, the effect of gefitinib was evaluated in H1975 cells harbouring EGFR-T790M. This mutation restores EGFR activity by increasing the affinity of the receptor for ATP, thereby competitively displacing reversible EGFR-TKIs, such as gefitinib [12]. As shown in the representative blots in Figure 5A, gefitinib did not affect migration or invasion of H1975 cells presumably due to its lack of effect on EGFR and Src phosphorylation (Figure 5B, C, D). In contrast, the second-generation inhibitors PD168393 and BIBW2992 (afatinib), which are able to covalently interact with Cys797 in the catalytic domain of EGFR, inhibited both EGFR and Src phosphorylation leading to the reduction of cell migration and invasion. Similar results were obtained in H1975 cells with novel irreversible inhibitors recently synthesized by our group [32].

### EGFR and MET independently control cellular migration via Src signaling

To unravel the contribution of different signaling pathways in the increased migratory capability, dasatinib (Src inhibitor), SU11274 (MET inhibitor), U0126 (ERK1/2 inhibitor), and NVP-BEZ235 (PI3K/mTOR inhibitor) were tested in HCC827 GR5 cells gefitinib-deprived for 7 days by Boyden chambers assay. 

**Figure 6:**
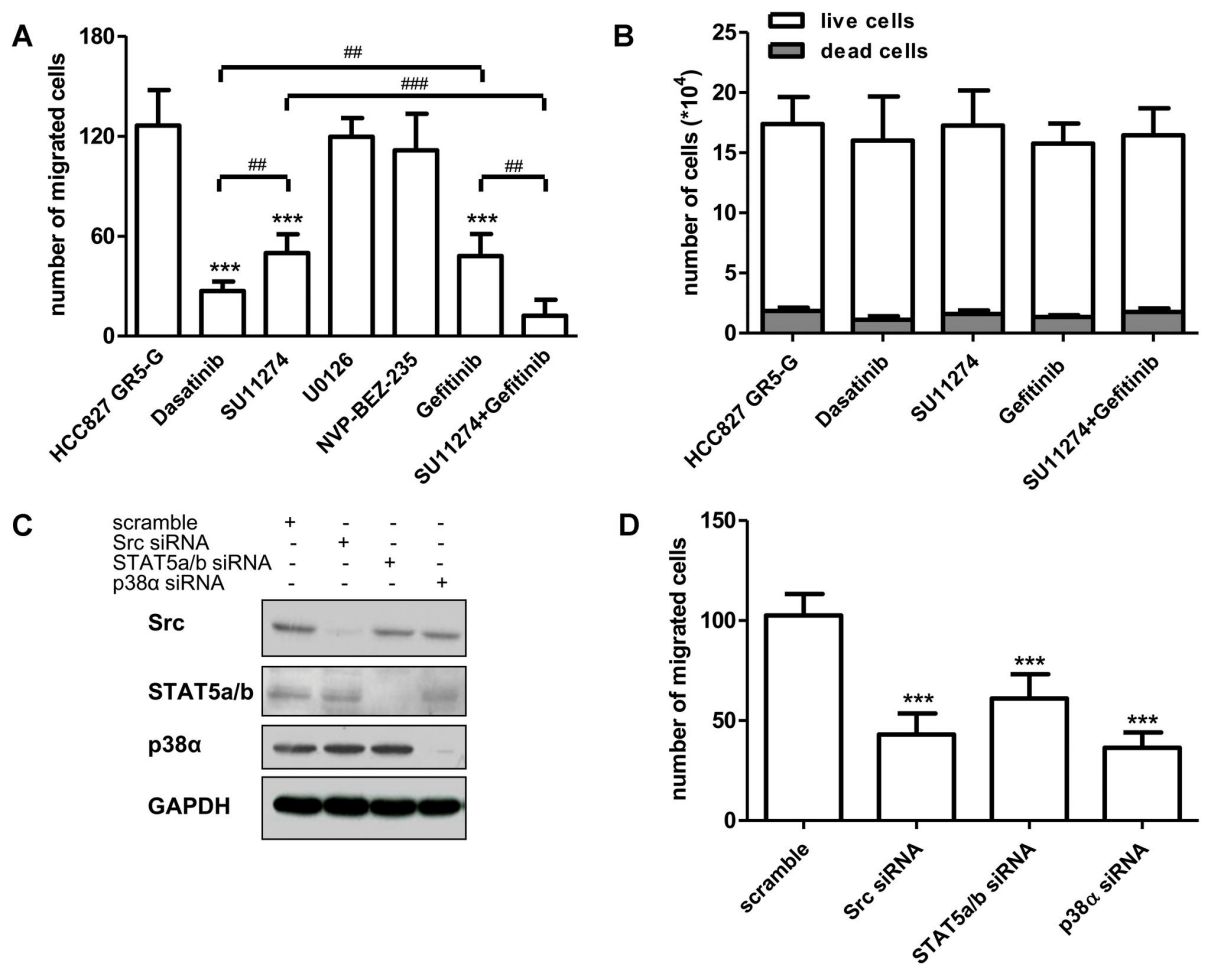
Effect of signal transduction pathways inhibition on cell migration of HCC827 GR5 cells deprived of gefitinib. (A) HCC827 GR5-G (maintained in the absence of gefitinib for 7 days) cells were exposed to dasatinib 0.01μM, SU11271 1μM, U0126 10μM, NVP-BEZ235 0.1μM, gefitinib 1μM or SU11271 1μM + gefitinib 1μM during migration time. Columns, means of 10 fields counted; bars, SD. Result is representative of three independent experiments. (B) HCC827 GR5-G cells were incubated with dasatinib 0.01μM, SU11271 1μM, gefitinib 1μM or SU11271 1μM + gefitinib 1μM. After 24h cells were counted and cell death was evaluated by fluorescence microscopy on Hoechst/PI stained cells. Columns, means of three independent experiments *, significance vs HCC827 GR5-G. ***P<0.001, **P<0.01; ^###^P<0.001, ^##^P<0.01. HCC827 GR5-G were transfected with Src, STAT5a/b, p38 siRNA or control siRNA (scramble) for 48 h. Then medium was replaced with fresh medium for 16h and the expression of the indicated proteins was analyzed by Western blotting (C) or cells were seeded on culture inserts for migration assay (D). Columns, means of 10 fields counted; bars, SD. Results are representative of three independent experiments. ***P<0.001.

Treatment with U0126 or NVP-BEZ235 did not affect migration of HCC827 GR5 cells deprived of gefitinib for 7 days. By contrast SU11274 inhibited cell migration with results similar to those achieved with gefitinib. Moreover, dasatinib and the combination of gefitinib with SU11274 almost completely suppressed migration (Figure 6A). The single drug concentrations used were able to completely suppress the phosphorylation of the respective targets (not shown), and the drugs that impaired cell migration, at the dose used, did not affect cell proliferation and cell viability at least until 24 hours (Figure 6B). These findings suggest that cell migration was controlled by both EGFR and MET through Src activation. 

In order to further confirm these results and the data shown in Figure 3C and 3D documenting up-regulation of Src, STAT5 and p38 after gefitinib deprivation, HCC827 GR5 cells maintained for 7 days in the absence of gefitinib were transfected with siRNAs targeting of Src, STAT5a/b or p38α. 

We found that knock-down of Src, STAT5a/b or p38α resulted in an almost complete suppression of expression of the corresponding proteins (Figure 6C) and significantly inhibited cell migration induced by gefitinib deprivation compared to the negative siRNA-scramble control (Figure 6D). 

### Gefitinib inhibits epithelial-mesenchymal transition

**Figure 7:**
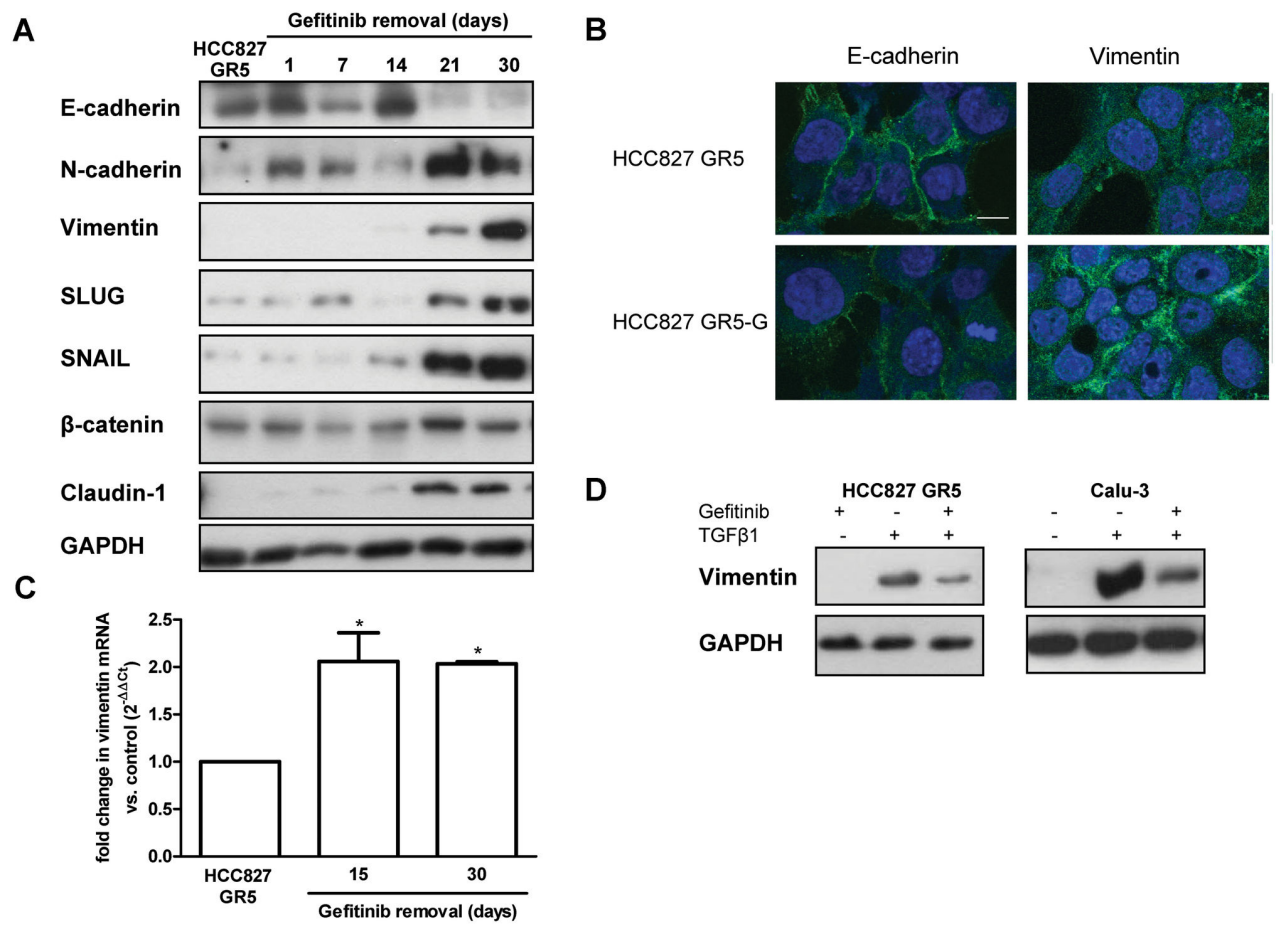
Effect of Gefitinib on EMT. (A) HCC827 GR5 cells were deprived of gefitinib for 1, 7, 14, 21 or 30 days. Expression of the indicated proteins was analyzed by Western blotting at each time point. Results are representative of three independent experiments. (B) Confocal immunofluorescence analysis of HCC827 GR5 and HCC827 GR5-G (maintained in the absence of gefitinib for 30 days) with antibody against E-cadherin and vimentin (green fluorescence). The nuclei were stained with Draq5 (blue fluorescence). Scale Bar: 10μm. (C) Comparison of vimentin mRNA by quantitative RT-PCR in HCC827 GR5 gefitinib-maintained cells versus gefitinib-deprived cells. The fold change was calculated using the 2^−ΔΔCT^ method relative to gefitinib-maintained cells used as control. (D) HCC827 GR5 cells were incubated with 2ng/ml TGFβ1 in the absence or in the presence of gefitinib 1μM. After 3 days, protein expression was assessed by Western blotting using the indicated antibodies. Results are representative of three independent experiments. *P<0.05.

To assess the involvement of EMT in the enhanced cell migration and invasion after gefitinib removal, mesenchymal associated features were evaluated. On the molecular level, EMT is defined by down-regulation of E-cadherin and increased expression of N-cadherin and vimentin. E-cadherin expression is regulated by the Wnt/β-catenin-mediated transcription of zinc-finger proteins such as SLUG and SNAIL [33]. As reported in Figure 7A, gefitinib removal induced a decrease in E-cadherin expression while the expression level of N-cadherin, vimentin and negative regulators β-catenin, SLUG and SNAIL were increased compared to cells continuously maintained with gefitinib. Moreover, an increase in the expression of the tight junction protein Claudin-1, recently reported to have a pivotal role in the induction of the EMT program, was also observed [34]. Unexpectedly, these markers of EMT appeared after 21 days of gefitinib removal whereas enhanced motility was observed already after 3 days (see Figure 3A). 

Immunofluorescence confocal microscopy confirmed the decrease of E-cadherin expression on the cell membrane and the increased levels of vimentin after 30 days of gefitinib removal (Figure 7B).

A significant increase of vimentin was also detected at gene expression level by qRT-PCR after 15 days of gefitinib removal as compared to gefitinib maintenance (Figure 7C). 

Since the transforming growth factor-β1 (TGF-β1) has been recently reported to induce EMT phenotype in human lung cancer cells [35,36] we analyzed whether gefitinib could inhibit TGF-β1-induced EMT. HCC827 GR5 and Calu-3 cells (a NSCLC cell line with epithelial features as previously reported [37]) were treated with 2 ng/ml TGF-β1 for 72 hours with or without 1µM gefitinib. As shown in Figure 7D, TGF-β1 treatment induced an increase of vimentin expression which was partially reverted by addition of gefitinib in the culture medium. 

These results confirm the involvement of TGF-β1 in the transforming process to mesenchymal phenotype and indicate the potential role of gefitinib in counteracting EMT after tumor progression.

## Discussion

One of the major findings of our study is that, independently from the effect on cell proliferation and growth, maintenance of gefitinib potentially limit the acquisition of a migratory and invasive phenotype in NSCLC cells characterized by *MET* amplification-driven resistance. We also demonstrated that gefitinib withdrawal leads to EGFR signaling reactivation, which is involved in the acquisition of cell aggressiveness behaviour. In particular, despite amplification of *MET*, whose migration/invasion-promoting activity is well established, our results suggest that persistent inhibition of EGFR is sufficient to maintain a low invasive phenotype in HCC827-GR5 gefitinib-resistant cells. These data support the role of EGFR in cell motility and invasiveness and suggest the importance of EGFR-TKIs maintenance treatment after tumor progression.

Of note, the continuous exposure of H1975 cells, carrying the T790M mutation, to gefitinib did not modify their migratory phenotype, possibly because of the low affinity of the mutated receptor for this drug. Indeed, irreversible EGFR-TKIs such as PD168393 or BIBW2992 significantly reduced H1975 cell migration and invasion, confirming the importance of EGFR in controlling these processes.

A number of studies investigated the cross talk between MET and EGFR demonstrating its contribution to cancer growth, migration and invasion [38–40]. Activation of these two receptors initiate similar signal transduction pathways including Src signaling. In HCC827 GR5 cells, only the combination of gefitinib with the MET-TKI PHA-665752 was shown to inhibit proliferation and to induce cell death. These cells were resistant to both gefitinib and PHA-665752 alone, indicating that either EGFR or MET signaling were essential to sustain cell proliferation and viability [13]. Conversely, the Src inhibitor dasatinib alone was able to overcome gefitinib resistance in this cell line [30].

Notably our results indicate that migration can be inhibited with the same efficacy by treating HCC827 GR5 cells with gefitinib or the MET inhibitor SU11274 as single treatment, suggesting their parallel and independent control in cell migration process. Combined inhibition of EGFR and MET resulted in a further inhibition of cell migration and a similar result was obtained with the Src inhibitor dasatinib.

Src and other members of its family of kinases such as Lyn or Fyn, exert a key role in cell migration and invasion through multiple downstream mediators including STAT, CAS, PI3K, FAK, paxillin, and others [41]. Some of these pathways emerged also in our studies, using a phospho-kinase array, that showed the active involvement of eight phosphorylated kinases (EGFR, p38α, Src, Lyn, STAT2, STAT6, STAT5a/b and c-Jun), in early processes associated with cell motility and invasiveness which can be modulated by gefitinib despite tumor progression. The functional role of Src, STAT5a/b and p38α proteins in modulating the migratory properties of HCC827 GR5 cells in response to gefitinib deprivation was further confirmed by siRNA-mediated silencing experiments.

STAT5a/b activation has been previously associated with increased migration and invasion, suppression of cell surface expression of E-cadherin and metastatic dissemination of prostate cancer cells [42]. In this study we demonstrated that in gefitinib-deprived HCC827 GR5 cells STAT5 was further phosphorylated at the phosphorylation site Tyr^694^ residue which is known to be associated with EGFR-dependent Src activation [43], whereas the JAK phosphorylation site Tyr^699^ was not modified. Significant modulation of other STAT family members, such as STAT2 and STAT6 was also observed in this study. STAT6 over-expression and activity have been previously reported to correlate with promotion of cell migration in prostate cancer cells [44] and invasiveness growth in glioblastoma [45]. Further studies are warranted to clarify the role of these proteins in cancer invasiveness.

The involvement of p38 in the modulation of cell motility and invasiveness through the regulation of MMPs has also been described [46]. In particular, members of the S100 family of calcium-binding proteins promoted cell migration and invasion through p38 MAPK-dependent NF-κB activation which increased MMP-2 and MMP-12 expression in gastric cells [47]. Moreover, baicalin suppressed cell migration and invasiveness in breast cancer MDA-MB-231 cells by down-regulating p38 MAPK pathway and consequently MMP-2 and MMP-9 expression [48]. In agreement with these and other previous studies, our results showed that gefitinib-treated NSCLC cells with MET amplification display a significant reduction in both MMP-2 and MMP-9 proteolytic activity associated with reduced phosphorylation of p38 MAPK. This modulation was detected by both phospho-kinase array and Western blot, and might be attributed to the direct effect of gefitinib.

There is a growing evidence that EMT contributes to invasive and metastatic tumor growth [49]. EMT is a complex process mainly characterized by down-regulation of markers commonly expressed in epithelial cells (e.g. E-cadherin), and increased expression of mesenchymal markers such as N-cadherin and vimentin. Src activation is a potent trigger for EMT induction [41], causing dissociation of the E-cadherin/β-catenin complex and degradation of E-cadherin by promoting its phosphorylation, ubiquitination, endocytosis and lysosomal degradation [50].

Importantly, we demonstrated that gefitinib maintenance after acquisition of resistance is essential to inhibit phenotypic changes associated with EMT. Moreover, gefitinib can prevent the EMT mediated by TGF-β1 and this might also control resistance to apoptosis and the emergence of stem cell like properties as described in previous studies on EMT [49].

In conclusion, we demonstrated that despite tumor progression after treatment with EGFR-TKIs, NSCLCs with *MET* amplification are still dependent on EGFR signaling. In these tumors EGFR plays an important role in cell motility and invasiveness and prompts the EMT process possibly via Src signaling. For all these reasons, the maintenance of gefitinib after tumor progression emerges as an important new therapeutic strategy to inhibit EGFR-mediated aggressive behaviour in NSCLC with MET amplification.
